# Exploring Chinese Consumers’ Attitudes Towards Pet Nutritional Products and Their Continuous Purchase Intentions: A Dual-Phase Analysis Using SEM and ANN

**DOI:** 10.3390/bs15030255

**Published:** 2025-02-23

**Authors:** Jiexiang Jin, Binbin Yang

**Affiliations:** Department of International Business and Commerce, Graduate School of Konkuk University, 120 Neungdong-ro, Gwangjin-gu, Seoul 05029, Republic of Korea

**Keywords:** pet nutritional products, health belief model, attitudes, continuous purchase intentions, SEM-ANN

## Abstract

As the role of pets evolves, they are increasingly regarded as members of the family. Although incapable of making independent decisions, pets become unique consumer groups through the purchases of specific products and services made by their owners. In China, the pet economy has developed into a novel economic sector. With the growing concern for pet health, the demand for pet nutritional products continues to rise. This study aims to explore Chinese consumers’ attitudes and intentions towards pet nutritional products in this burgeoning market. A survey was carried out on 600 Chinese consumers who had purchased pet nutritional products in 2024. A two-stage analysis using structural equation modeling and artificial neural network examined the correlation within the research model across 506 samples. The results indicate that perceived benefits, perceived severity, perceived susceptibility, and health consciousness positively influence attitudes towards pet nutritional products, while perceived barriers, health risks, and economic risks negatively impact attitudes. The attitudes of consumers significantly enhance their continuous purchase intentions. This study lays an essential groundwork for advancing pet food products, refining marketing approaches, and conducting future research.

## 1. Introduction

Pets serve not only as an organic bridge through which humans can better integrate with nature but also play a constructive role in daily human life (Scoresby et al., 2021). For instance, pet ownership aids in the management and alleviation of stress (Rhoades et al., 2015), yielding benefits for both mental and physical health (Wu et al., 2018). With the rise in national income levels in China and changes in the demographic structure, there has been a growth in the population of pet owners, alongside an evolution in the roles pets play and the pet-rearing philosophies upheld by their owners, who increasingly aspire to higher quality standards (Li et al., 2024).

According to data from 2023, the number of domestic pets (dogs and cats) in urban China has surpassed 120 million, with cats accounting for 69.8 million and dogs amounting to 51.75 million. The demographic characteristics of pet owners are becoming increasingly distinct. Notably, individuals born in the 1990s and 1980s constitute the primary cohort of pet owners, representing 46.6% and 31.1% of the total, respectively. Following them are those born in the 2000s at 10.1%, the 1970s at 8.1%, and pre-1970s at 4.1%. Therefore, pet ownership has gained widespread acceptance across various age groups, and its influence continues to expand (2023–2024 China pet nutrition products consumption report, 2023). Moreover, the demand for pets has spurred the growth of the pet industry, which has emerged as a new economic form in China (Zhang et al., 2022). According to the 2024–2025 China pet industry operation status and consumer market monitoring report (2024), the pet industry in China is in a phase of rapid expansion. In 2023, the market size reached CNY 592.8 billion, with an expected increase to CNY 811.4 billion by 2025. Consumer spending is primarily concentrated on food, supplies, and healthcare. Particularly in the domain of pet food, consumers are increasingly recognizing the close relationship between pet nutrition and health (Barrett-Jolley & German, 2022). Studies have shown that pet nutritional products can promote health and alleviate diseases (R. C. Gupta et al., 2021), regulate gastrointestinal function (Atuahene et al., 2024), and improve the quality of skin and fur (Burron et al., 2024), thereby significantly driving up the demand for pet nutritional products. It is reported that, in 2023, the market size for pet nutritional products in China reached CNY 7.22 billion, a rise of CNY 1.52 billion from 2022 (2023). Research by Xue et al. (2022) indicates that future development of pet food in China will progress towards health-oriented and functional products, with pet owners playing a crucial role in the pet food market (Xiao et al., 2021). As the market size of pet nutritional products in China continues to expand, studying the factors influencing Chinese consumers’ purchasing behaviors becomes particularly significant.

Despite the growth in the market size of pet nutritional products, research on consumer behavior and decision-making processes lags behind, often skewed towards product functionality or ingredient-based studies. For instance, pet foods enriched with components such as Omega-3 (Johnson et al., 2020), prebiotics (R. Kumar & Sharma, 2024), and microalgae (Vasconcellos et al., 2023) have been shown to improve pet health. As consumer attention to pet health increases and the demand for pet nutritional products rapidly grows, the diversity of consumer needs for these products also increases (2024–2025 China pet industry operation status and consumer market monitoring report, 2024). However, existing research lacks a thorough exploration of the purchasing motives, behavioral patterns, and psychological processes of consumers when selecting such products. Therefore, it is necessary to integrate relevant theories and variables to study the attitudes of consumers towards pet nutritional products and their intentions to continue purchasing them.

There is limited research on the determinants of attitudes towards purchasing pet nutritional products. We intend to utilize factors influencing consumer food purchases, as the trend of pet humanization encourages pet owners to align their pet food selections with their own food choices (White, 2023). The Health Belief Model (HBM) is one of the most widely recognized and accepted theories within the domain of health behavior (Rosenstock, 2005). Research conducted by Bauer (2004) delineates how individual health beliefs influence preventative behaviors, elucidating the relationship between health beliefs and health actions. This model has been applied to various contexts, such as vaccination uptake (Limbu et al., 2022; Ibrahim et al., 2023), retrieval and sharing of health information (Malik et al., 2023), and mobile health technologies (mHealth) (Zahed et al., 2023). Furthermore, Glick et al. (2024) have utilized the HBM to explore consumer intentions to purchase nutritional food products. Attitudes also play a crucial role in influencing consumers’ purchasing behaviors concerning functional foods (Sukboonyasatit, 2009), a point substantiated by the study from Rezai et al. (2017), which incorporated attitudes into the purchasing intention–HBM connection. Consequently, the HBM is utilized for analyzing consumer attitudes toward pet nutrition products and investigating how these attitudes further influence their purchasing intentions.

It is important to consider how individuals perceive health threats and decide whether to engage in preventative behaviors (Ataei et al., 2021). Perceived risk involves an individual’s assessment of potential negative outcomes in specific purchasing or behavioral decisions (Jacoby & Kaplan, 1972). In different decision-making situations, if consumers are aware of the uncertainty of the result of the purchase decision or worry that the purchase behavior may lead to adverse consequences, they will have the perceived risk (PR) of purchase (Huo & Liu, 2022). Consumers have different dimensions of PR for different commodities, and different dimensions of PR have different impacts on consumers’ behavioral intentions (R. Liu et al., 2014). The PR of food is an important variable affecting attitude (Xie et al., 2020). Therefore, this study aims to explore the PR factors of pet nutritional food and further analyze its impact on attitude. At the same time, health consciousness (HC) has a significant impact on consumers’ decision-making process and behavior (Elsotouhy et al., 2024). HC is also an important factor in determining consumers’ attitude towards food (Michaelidou & Hassan, 2008; Nagaraj, 2021). Therefore, in the context of purchasing pet nutritional products, incorporating PR and HC into the HBM can enhance our understanding of consumer attitudes and their intentions to continue purchasing.

The research results can provide a scientific basis for consumer education, help consumers make more rational choices when buying pet nutrition food, and bring practical value to business operations. Through in-depth insights into consumer behavior, companies can more accurately acquire potential customers, thus providing data support and decision-making reference for strategic planning of the pet nutrition food market. This study employs a combined analysis method of Structural Equation Modeling (SEM) and Artificial Neural Networks (ANN), which, unlike traditional linear hypothesis-based studies, can capture both linear and non-linear relationships (Leong et al., 2024). This approach allows for a more comprehensive and precise prediction of consumer behavior, offering a novel perspective for future research into the pet nutritional product market. Ultimately, this will help enhance pets’ health and quality of life.

## 2. Theoretical Background and Hypothesis Development

### 2.1. The Health Belief Model

The HBM, founded on the principles of expected value theory, was developed in the late 1950s as a model for explaining individuals’ preventive health behaviors (Rosenstock, 2005). The HBM conceptualizes cognitive factors as predictors of individual health-related behaviors and posits two primary sets of beliefs: perceived threat, which includes both perceived susceptibility (PSUS) and perceived severity (PSEV), and behavioral evaluation, which encompasses perceived barriers (PBA) and perceived benefits (PB) (Rosenstock, 1974; Witte, 1992). Extensive research demonstrates the widespread application of the HBM in the domain of food-related behaviors (M. Wang et al., 2021; Y. Wang et al., 2022; Riazi et al., 2024).

Within the framework of the HBM, PSUS refers to an individual’s subjective perception of the risk of acquiring a disease or health condition (Rosenstock, 1974, 2005). In this study, PSUS is defined as an individual’s subjective belief in the likelihood of pets contracting various diseases. PSEV is the individual’s subjective assessment of the potential serious consequences of contracting a disease (Rosenstock, 1974, 2005). Herein, PSEV is defined as the individual’s cognition of the severity of outcomes resulting from pets becoming ill. PB refer to the believed advantages or efficacy of health behaviors in reducing the threat of disease (Rosenstock, 1974, 2005). In the current research, PB are defined as the individual’s perception of the positive impacts or advantages of using pet health food. PBA denote the individual’s perceived difficulties or obstacles encountered when engaging in a particular health behavior (Rosenstock, 1974, 2005). In this study, PBA are defined as the subjective perceptions of challenges or impediments consumers face when choosing and utilizing pet health foods.

The intention to purchase food is determined by attitudes (Jahn et al., 2019). The study by Rezai et al. (2017) on the intention to purchase natural functional foods utilized the HBM to analyze the relationship between attitudes and purchase intentions. Prior research supports the inclusion of attitudes as a meaningful variable in the relationship between the HBM constructs and purchasing behavior.

Research by Rezai et al. (2017) indicates that PSUS and PB positively influence attitudes, while PBA have a negative impact. Studies by Zhao et al. (2018) on Mobile Health Services and Prasetyo et al. (2020) on disease prevention demonstrate positive impacts of PSUS and PSEV on attitudes. Furthermore, research by Erkan et al. (2021) on genetically modified foods shows that PB positively affect attitudes. Previous studies across various topics validate that PSUS, PSEV, PB, and PBA are appropriate theoretical constructs for explaining attitudes.

Based on these findings, we propose the following hypotheses:

**H1:**  *PSUS positively influences attitudes towards pet nutritional foods.*

**H2:**  *PSEV positively influences attitudes towards pet nutritional foods.*

**H3:**  *PB positively influence attitudes towards pet nutritional foods.*

**H4:**  *PBA negatively influence attitudes towards pet nutritional foods.*

### 2.2. Perceived Risk

Consumers, when considering the purchase of products or services, also weigh the potential losses they might incur, thereby experiencing PR (Roselius, 1971). PR is extensively utilized in the domains of marketing and psychological research to explore consumer purchasing behavior (Zhu & Deng, 2020). As consumers may avoid selecting certain foods due to PRs (Choi et al., 2013), their attitudes towards food are significantly influenced by these PRs (Loh & Hassan, 2021). Consequently, in predicting consumer attitudes and behavioral intentions, PR should be regarded as a key variable. Here, PR is defined as the degree of uncertainty or potential loss perceived by consumers when purchasing pet nutrition products.

PR is multidimensional, and Jacoby and Kaplan (1972) categorize PR into five dimensions: economic, functional, social, health, and psychological. Subsequent academic research on consumer PR has largely been conducted along these dimensions. The concerns consumers have regarding food consumption are influenced by a multitude of issues, and understanding how people perceive these risks is crucial for comprehending how they cope with the potential risks associated with their choices (Choi et al., 2013). Health risks due to food safety (Choi et al., 2013; Huo & Liu, 2022; Zhang et al., 2024), economic risks (ERs) when the price of food exceeds its perceived value (Huo & Liu, 2022; Pillai et al., 2022), and social risks (SRs) associated with potential loss of status within social groups (Choi et al., 2013) have become prevalent concerns in food purchasing. In this study, the perceived risks associated with pet nutritional products are categorized into three dimensions: health risk (HR), economic risk (ER), and social risk (SR).

First, health risk (HR) refers to the extent to which consumers are concerned that pet nutritional products may negatively impact their pets’ health. Economic risk (ER) pertains to consumers’ concerns about the possibility of financial loss due to pet nutritional products being priced higher than their perceived value. Social risk (SR) involves consumers’ worries that their choices of pet food may be judged or negatively perceived by their social groups.

Choi et al. (2013) found in their study on street food purchases that social and health risks had a significant negative impact on attitudes. Pillai et al. (2022) observed that ERs negatively affected consumer attitudes in online food purchases. V. Gupta and Duggal (2020) noted significant negative impacts of social and ERs on attitudes in their study of online food delivery applications. An et al. (2024) reported that health risks negatively influenced consumer attitudes in their research on culinary tourism.

Based on these findings, the following hypotheses are proposed:

**H5:**  *Health risks will have a negative impact on attitudes towards pet nutrition products.*

**H6:**  *ERs will have a negative impact on attitudes towards pet nutrition products.*

**H7:**  *SRs will have a negative impact on attitudes towards pet nutrition products.*

### 2.3. Health Consciousness

Health consciousness (HC) refers to an individual’s intrinsic preference or consumer characteristic that influences behavior related to health matters (Elsotouhy et al., 2024; Chen & Lin, 2018). Consumers who are health-conscious typically pay close attention to their health status and take proactive measures to either improve or maintain their health (Michaelidou & Hassan, 2008). In other words, the intention or motivation of an individual to engage in health behaviors directly affects the alteration of their health-related actions (G. A. Kumar, 2021). In this study, HC is defined as the degree of a consumer’s concern for pet health status and the extent to which they take active steps to maintain and improve health.

Talwar et al. (2021) found in their study on natural foods that HC has a significant positive effect on attitudes towards natural foods. Similarly, Kaur et al. (2023) observed a positive influence of HC on attitudes towards foods marketed with enhanced immunity claims. Moreover, Leblebici Kocer et al. (2023) discovered that HC positively impacts attitudes towards organic foods.

Based on these findings, we propose the following hypothesis:

**H8:**  *HC has a positive effect on attitudes towards pet nutritional foods.*

### 2.4. Attitude and Continuous Purchase Intentions

Attitude is a learned predisposition to respond consistently in a favorable or unfavorable (positive or negative) manner to a given situation (Fishbein & Ajzen, 1977). Consequently, attitudes influence behavioral intentions, which in turn affect actual behaviors (Ajzen, 1991). In this study, attitude is defined as the consumer’s positive or negative reactions to the use of pet health functional foods.

Continuous purchase intention (CPI) is defined as the consumer’s intention to repeatedly buy a product or service under the condition of continued preference, even when faced with marketing efforts for other products or services (Oliver, 1980). From a business perspective, the continuance usage by consumers is a key success factor in a rapidly changing business environment and amidst intense competition among brands (Garbarino & Johnson, 1999; Wibowo et al., 2021). In this study, CPI is defined as the intention of consumers to continue purchasing a pet health functional food product after the initial purchase.

Lim and An (2021) found in their study on health supplements that a positive attitude towards health supplements increased the intention to purchase. Similarly, Y. Khan et al. (2022) showed that attitudes have a positive impact on the purchase intentions for organic foods. Qi et al. (2023) confirmed in their study on organic foods that attitudes positively influence the CPIs for organic foods. Sahranavard et al. (2024) confirmed these findings in their study on online food ordering services, demonstrating that the level of consumer attitudes positively influences repurchase intentions.

Based on these findings, we propose the following hypothesis:

**H9:**  *Attitudes towards pet nutritional foods positively influence CPIs.*

### 2.5. Research Model

**Figure 1:**
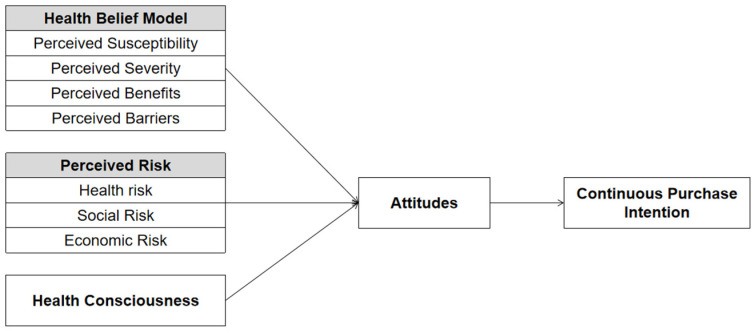
Research model.

As depicted in Figure 1, this study examines the effects of PSUS, PSEV, PB, PBA, PRs, and HC on attitudes, and explores the impact of attitudes on CPIs.

## 3. Research Method

### 3.1. Data Collection

To test the proposed hypotheses, an online survey was conducted using quota sampling. This study targeted Chinese consumers who had purchased pet nutritional products in the past year, ensuring a balanced distribution of respondents based on key demographic factors such as age, gender level. The data were collected from 16 December to 29 December 2024, using the Chinese online survey platform WJX (https://www.wjx.cn/, accessed 16 December 2024). To ensure respondents understood the concept of pet nutritional products clearly, the survey included definitions and examples of such products. Based on calculations from G*Power version 3 software (Faul et al., 2009), the minimum sample size required for the study was determined to be 118. A total of 600 surveys were administered, and after excluding invalid responses, 506 valid questionnaires were obtained, resulting in a response rate of 84.3%, which exceeds the required minimum sample size. The demographic characteristics of the respondents are presented in Table 1.

### 3.2. Questionnaire Design

This study utilized scales for assessing health-functional foods for pets, as shown in Table 2. The scales employed have been validated for reliability and validity in previous research and were appropriately modified to meet the needs of this study. Questions related to the variables of the HBM were based on the studies by Rezai et al. (2017) and Rosenstock (2005). Questions concerning PR were derived from the research designs of Choi et al. (2013), Huo and Liu (2022), and Zhang et al. (2024). Issues regarding HC were referenced from research by Chen and Lin (2018), while questions on attitudes originated from Oliver (1980). Questions pertaining to CPI were based on the study by Y. Khan et al. (2022). Sociodemographic variables included gender, age, level of education, and average monthly total income. Sociodemographic variables were measured using a nominal scale, while the remaining variables were assessed using a 5-point Likert scale, where 1 indicates “strongly disagree” and 5 indicates “strongly agree”.

### 3.3. Data Analytic Procedure

The study employed a two-stage approach using Partial Least Squares Structural Equation Modeling (PLS-SEM) and Artificial Neural Networks (ANN). In the first phase of the analysis, PLS-SEM method was applied using SmartPLS software version 4.0 to test the proposed conceptual model. PLS-SEM is known for its superiority in testing complex models and in theoretical construction compared to covariance-based structural equation modeling (CB-SEM) (Ooi & Tan, 2016; Ooi et al., 2020). Given that SEM primarily relies on linear models, yet human behavior is inherently non-linear (Almufarreh, 2024), structural equation modeling typically assumes compensatory relationships among variables, where the deficiency in one variable can be offset by the strong influence of others. However, non-compensatory relationships challenge this assumption (Leong et al., 2024). In the second stage of analysis, ANN analysis was employed to supplement the SEM analysis. Compared to SEM, ANN holds significant advantages in predicting non-linear and non-compensatory relationships (Tewari et al., 2022). Furthermore, ANN is not constrained by multivariate assumptions, which affords greater flexibility in complex data modeling (Leong et al., 2024). Therefore, by adopting a hybrid SEM-ANN approach, this study is able to meet both hypothesis testing and prediction needs effectively.

## 4. Data Analysis and Results

### 4.1. Common Method Bias

Given that the study employed a cross-sectional design, potential threats of common method bias were assessed using procedural and statistical methods (Tan & Ooi, 2018; Lee et al., 2022). Procedurally, respondents were informed that there were no right or wrong answers and assured of the confidentiality and anonymity of their responses. Statistical exploration of common method bias (CMB) was conducted using the method proposed by Liang et al. (2007). The results, displayed in Table 3, showed that the average substantive variance was 0.671 while the method variance averaged 0.003, yielding a ratio of approximately 238:1 between substantive and method variance. Furthermore, most method factor loadings were not significant. Thus, this analysis indicates that common method bias does not pose a substantial issue within the scope of this study.

### 4.2. Reliability and Validity Test

The Cronbach’s alpha values ranged from 0.733 to 0.831, and composite reliability (CR) ranged from 0.849 to 0.888, both exceeding the threshold of 0.7 suggested by Fornell and Larcker (1981), indicating satisfactory reliability for all latent variables. The average variance extracted (AVE) ranged from 0.608 to 0.725, and item loadings (IL) ranged from 0.716 to 0.880, both surpassing the recommended minimum of 0.5 by Fornell and Larcker (1981) and Hair et al. (2011). Thus, the reliability and validity conditions of the constructed measures are satisfactory. The detailed results are presented in Table 4.

Additionally, the Fornell–Larcker criterion was applied, and Table 5 displays the correlation coefficients between variables alongside the square roots of AVE values (shown on the diagonal). The results demonstrate that the square roots of AVE are higher than the correlation coefficients between constructs, confirming satisfactory discriminant validity (Hew et al., 2017).

### 4.3. Structural Model and Hypotheses Testing

All Variance Inflation Factor (VIF) values were below the threshold of 3, indicating no significant issues with multicollinearity (Wong et al., 2020). In this study, the R^2^ value for ATT was 0.607, and for CPI, it was 0.427. In consumer research, an R^2^ value exceeding 0.20 is considered to have practical significance (Sarstedt et al., 2022; Bakır & Itani, 2024), thus the results herein provide satisfactory explanatory power. Moreover, to assess the model fit, the Standardized Root Mean Square Residual (SRMR) was employed. The SRMR value was 0.048, which is below the recommended threshold of 0.08 (Latan et al., 2019), indicating that the model fit is acceptable.

Table 6 summarizes the results of hypothesis testing. Hypotheses H1 to H4 examined the impact of HBM variables on ATT. PSUS (β = 0.161, *p* < 0.001), PSEV (β = 0.127, *p* < 0.001), and PB (β = 0.172, *p* < 0.001) all had a significant positive impact on ATT, whereas PBA (β = −0.141, *p* < 0.001) had a significant negative impact. These results support hypotheses H1, H2, H3, and H4. These findings are consistent with the assumptions of the HBM, where PSUS, PSEV, and PB positively influence attitudes towards pet nutrition, while PBA negatively affects these attitudes.

Social Risks (SR) (β = −0.052, *p* > 0.05, ns) had no significant impact on the attitude toward purchasing pet nutrition products, thus hypothesis H7 was rejected. However, HRs (β = −0.106, *p* < 0.01) and ERs (β = −0.211, *p* < 0.01) both had significant negative impacts, supporting hypotheses H5 and H6, respectively. These results indicate that HR and ER are the primary factors limiting positive attitudes towards pet nutrition products.

Furthermore, HC had a positive impact on ATT (β = 0.227, *p* < 0.001), supporting hypothesis H8. ATT had a positive impact on CPI (β = 0.654, *p* < 0.001), supporting hypothesis H9.

To evaluate the out-of-sample predictive relevance of the model, we employed the PLSpredict procedure by Shmueli et al. (2019). As shown in Table 7, the Q^2^ predictive values for endogenous variables were significantly above zero. Additionally, the Root Mean Square Error (RMSE) values predicted by PLS-SEM were lower than those of the Linear Model (LM) baseline. Thus, the research model exhibits high predictive accuracy (Tiwari et al., 2024).

### 4.4. Analysis Using Artificial Neural Network (ANN)

**Figure 2:**
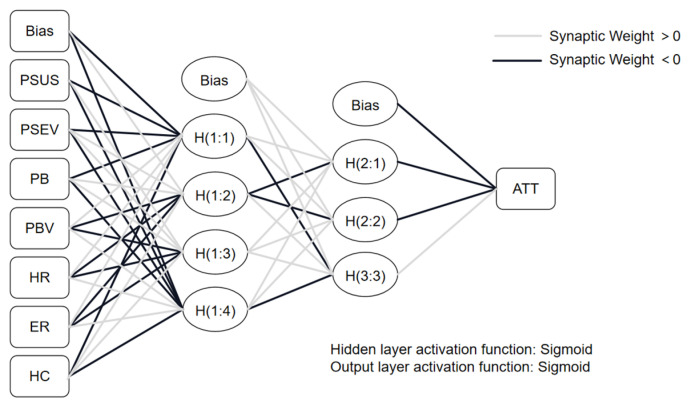
ANN model.

In this research, an ANN model was constructed utilizing the Multilayer Perceptron neural network architecture in SPSS 26 (refer to Figure 2). The ANN model facilitated the determination of the relative importance of various variables and reinforced the conclusions derived from the Structural Equation Modeling (SEM) (Dang et al., 2023). To assess the predictive capability of the model, ten-fold cross-validation was employed. Specifically, the dataset was randomly divided into ten equal parts, with 90% being used for training and the remaining 10% for testing (Leong et al., 2024). The accuracy of the ANN model across both training and test datasets was evaluated using the RMSE values (Lo et al., 2022), as shown in Table 8. Given that all RMSE values were negligible, the ANN model demonstrated excellent goodness of fit (Leong et al., 2020).

To gauge the predictive strength of each predictor, a sensitivity analysis (Non-Linear Relationship) was conducted to ascertain their normalized importance (Ng et al., 2022). Normalized importance was calculated by comparing the mean value of each predictor with the highest mean value, expressed as a percentage (Leong et al., 2024). The mean values and normalized importance of all predictors used in the ANN modeling process are detailed in Table 9. According to the results of the sensitivity analysis, ER (100%) was identified as the most prominent predictor of ATT in pet nutrition foods, followed by HC (98.19%), PSEV (76.75%), PSUS (74.43%), HR (74.21%), PB (72.17%), and PBA (69.74%).

## 5. Conclusions and Discussion

### 5.1. Theoretical Implications

Considering the scholarly significance of this study, it is pertinent to note that previous research predominantly utilized the HBM to analyze behavioral intentions through constructs such as PSUS, PSEV, PB, and PBA. Following recommendations from prior researchers, this study integrates attitudes into the HBM framework (Rezai et al., 2017). Consequently, attitudes were incorporated into the model examining the relationship between HBM and CPIs, thus underscoring the importance of attitudes. The findings indicate that PSUS, PSEV, PB, and PBA significantly influence attitudes, thereby providing a theoretical foundation for subsequent research.

Furthermore, this investigation not only analyzed the relationship between HBM variables and attitude but also examined the influence of various dimensions of PRs on attitude. Following the findings of earlier researchers, PRs were categorized into HRs, SRs, and ERs; the specific impacts of these categories on attitude were explored (Choi et al., 2013; Huo & Liu, 2022; Zhang et al., 2024). The findings reveal that higher perceived health and ERs associated with pet nutrition products correlate with more negative attitudes. This insight offers a meaningful theoretical basis for future typologies of PRs in pet nutrition products.

Thirdly, to enhance the understanding of the mechanisms behind attitude formation towards pet nutrition products, this study introduced HC as a key variable (Kaur et al., 2023). The results demonstrate that HC significantly affects attitudes towards pet nutrition products. This finding not only enriches the existing literature on pet nutrition products but also extends the range of known factors influencing consumer attitudes in this field. Additionally, the relationship between consumer attitudes towards pet nutrition products and their CPIs was analyzed, revealing the pivotal role of attitude in purchasing decisions.

Lastly, methodologically, this study employed the Structural Equation Modeling-Artificial Neural Network (SEM-ANN) analysis method. Compared to traditional approaches that assume linear relationships, SEM-ANN is capable of capturing both linear and compensatory as well as non-linear and non-compensatory relationships, providing a more comprehensive and in-depth analytical perspective for research in the relevant fields.

### 5.2. Practical Implications

In recent years, the pet economy in China has evolved into a novel economic sector (Zhang et al., 2022). Coinciding with a shift in pet-rearing philosophies, there has been a gradual elevation in pet owners’ consumption values towards a preference for high-quality products (Li et al., 2024). Consumers are increasingly recognizing the link between pet nutrition and health (Barrett-Jolley & German, 2022). However, prior research on pet nutrition primarily focused on the added nutritional components (Johnson et al., 2020; R. Kumar & Sharma, 2024; Vasconcellos et al., 2023), with relatively few studies investigating consumer purchasing intentions. Consequently, firms lack effective reference points for formulating market strategies. This study, therefore, provides critical insights for the pet nutrition industry, offering profound implications for business operators and market strategists to better understand consumer needs and purchasing motivations. Moreover, it supports further development within the pet economy.

Initially, PSUS, PSEV, PBA, and PB are pivotal factors shaping consumer attitudes towards pet nutritional products. Consistent with the findings of Zhao et al. (2018) in the mobile health sector, both PSUS and PSEV positively influence attitudes. This underscores the importance of threat assessment as a prerequisite for attitude formation (i.e., whether to adopt pet nutritional products) (Barrett-Jolley & German, 2022). Consequently, companies in the pet nutrition industry can foster a clearer understanding by producing intuitive educational materials, such as short videos and graphic content, which directly address the HRs associated with unhealthy dietary habits, such as obesity, diabetes, and dermatological issues. In line with Rezai et al.’s (2017) discoveries in the study of natural health functional foods, this research also indicates that PBA negatively impact consumer attitudes. Factors such as packaging design (Kwak & Cha, 2021) and the convenience of purchase (Z. Liu, 2024) are crucial in influencing consumer attitudes. Therefore, pet nutrition companies should optimize product packaging design and expand diverse purchasing channels. By providing clear and comprehensible packaging information, companies help consumers understand the product’s functions and usage methods. This effectively reduces the learning costs and usage barriers, thereby enhancing consumer acceptance and satisfaction. Furthermore, consistent with the findings of Erkan et al. (2021) in the domain of genetically modified foods, PB have a positive effect on attitudes. Given the importance of the benefits and improvements brought about by food (Rezai et al., 2017), pet nutrition companies should actively showcase the efficacy of their products through various media channels. They should also encourage existing customers to share their experiences with others or on social media, thereby enhancing the appeal of pet nutritional products.

Secondly, considering that PR can significantly influence individuals’ attitudes, decision-making processes, and behaviors (Loh & Hassan, 2021), this study investigates the impact of PR on attitudes towards pet nutrition products. Our findings indicate that SRs do not significantly affect usage attitudes. This suggests that although SRs are important, consumers evaluate their attitudes towards pet nutrition products based on the actual health status of their pets, rather than the opinions of others, thereby substantially diminishing the influence of SRs on their attitudes. Conversely, consistent with the research conducted by Choi et al. (2013) in the context of street food purchasing, this study identifies a negative impact of HRs on attitudes towards pet nutrition products. Concerns over food safety often lead to negative perceptions of the products (An et al., 2024). Therefore, establishing safe and reliable food production processes is crucial for enterprises in the pet nutrition industry. Firms should mitigate consumer concerns about food health and hygiene by making their production processes transparent and publicly displaying the manufacturing and quality control stages of pet nutrition products. As demonstrated by Pillai et al. (2022) in their study on online food purchasing, financial risks significantly influence consumer attitudes towards pet nutrition products. Consumers of pet nutrition products often harbor negative views due to uncertainties concerning product prices or cost-effectiveness. Consequently, enterprises should adopt reasonable pricing strategies to reduce economic uncertainties faced by consumers. These measures can effectively manage PRs and cultivate a positive attitude towards the use of pet nutrition products, thereby attracting more consumers.

Third, consumers’ HC has a significant positive impact on attitudes, a finding consistent with the findings of Leblebici Kocer et al. (2023) on organic foods. Therefore, pet nutrition food enterprises should enhance consumers’ HC through effective publicity strategies. Through the promotion of pet health knowledge, it can not only help consumers deepen their understanding of pet health, but also further enhance their HC, so as to improve their attitude towards pet nutrition food.

Fourth, attitudes toward pet nutritious food have a significant positive impact on continuous purchase intent. This finding is consistent with the conclusion of Qi et al. (2023) in the study of organic food. Enhancing consumer attitudes has been shown to be an effective means of promoting buyback intentions (Sahranavard et al., 2024). Therefore, pet nutrition food companies should establish a sound consumer feedback mechanism, timely listen to and solve consumer needs and concerns, in order to promote the continuous optimization of products. At the same time, promotional efforts should be increased to ensure that consumers fully understand the functions and purchase channels of pet nutritional food. In addition, it is critical to strengthen food safety measures and implement reasonable pricing in line with product value. By highlighting the unique advantages of pet nutrition food, demonstrating high standards of food safety and fair and transparent pricing strategies, positive user attitudes can be cultivated and consumers’ intention to continue to purchase pet nutrition food can be further enhanced.

Finally, financial risk and HC are key factors influencing attitudes towards nutritious food for pets. Enterprises should give priority to developing reasonable pricing strategies and strengthening the publicity of pet health knowledge to effectively improve consumers’ attitude towards pet nutrition food.

### 5.3. Limitations and Future Research

This study has identified several limitations. Firstly, while it has provided valuable insights based on the HBM and has offered a novel perspective on the relationship between attitudes and sustained purchasing intentions, it must be acknowledged that there are notable limitations. Specifically, due to the relatively scant literature on pet health food, this study did not examine the moderating variables between attitudes and sustained purchasing intentions. Future research should build upon this foundation by incorporating moderating variables such as trust, knowledge and perceived price, subjective norms, and product involvement (Sultan et al., 2020; K. Khan et al., 2022), which would allow for a more thorough exploration of the interplay between attitudes and sustained purchasing intentions.

Secondly, the variety of pet nutritional products is extensive, including those that promote health and alleviate diseases (R. C. Gupta et al., 2021), regulate gastrointestinal functions (Atuahene et al., 2024), and improve the quality of skin and fur (Burron et al., 2024). As the types vary, so do attitudes and the CPI. Future studies should conduct comparative research on these factors across different types of pet nutritional products.

Thirdly, this study primarily focuses on overall consumer behavior patterns rather than the impact of specific pet types. Consequently, it does not differentiate between types of pet owners, such as dog owners or cat owners. However, consumer attitudes and CPI may exhibit heterogeneity across different pet types. Future research could incorporate pet type as a control variable at the sample design stage to more accurately reveal the consumption attitudes and sustained purchasing intentions of various pet owner groups.

Fourthly, the participants of this study were all experienced consumers, thus precluding an examination of the reasons why potential users might choose not to purchase pet nutritional products. Future research should include participants who have never used pet nutritional products to more comprehensively explore the reasons for non-use, thereby providing more comprehensive insights into the field.

Finally, the sample of this study was limited to users in China. Although the findings offer valuable insights into the Chinese market, their applicability to other cultural and environmental contexts has not been confirmed. To validate these findings and better understand how these variables operate across different cultural backgrounds, future research should adopt a multicultural approach and include a broader range of participants for a more comprehensive analysis.

## Figures and Tables

**Table 1:** General characteristics of survey participants.

Classification	Indicators	Frequency	%
Gender	Male	257	50.8
	Female	249	49.2
Age	20–29	121	23.9
	30–39	131	25.9
	40–49	128	25.3
	Over 50 (including 50 years old)	126	24.9
Education level	High school or below	103	20.4
	University or college graduate	268	53.0
	Postgraduate or above	135	26.7
Monthly income	Less than CNY 6000 (excluding CNY 6000)	243	48.3
	Between CNY 6000 and CNY 9000 (excluding CNY 9000)	150	29.6
	More than CNY 9000	113	22.3

**Table 2:** Scale construction.

Constructs	Items	Item Source
Perceived Susceptibility (PSUS)	I believe pets are susceptible to health issues.	(Rezai et al., 2017; Rosenstock, 1974)
	I believe pets may develop health issues if no preventive measures are taken.	
	I believe an unbalanced diet contributes to health problems in pets.	
	I believe that pets, even if appearing healthy, may harbor latent diseases.	
Perceived Severity (PSEV)	Illness in pets can cause considerable inconvenience to family members.	
	Once ill, pets may have difficulty recovering.	
	Illness in pets may affect their lifespan.	
	Illness in pets may impair their normal daily activities.	
Perceived Benefits	Nutritional food for pets can reduce the risk of illness.	
	Nutritional food can improve the overall physical condition of pets.	
	Nutritional food for pets can prevent certain diseases (e.g., heart disease).	
Perceived Barriers	I am concerned that pets might dislike the taste of nutritional food.	
	I am unsure about how to select appropriate nutritional food for pets.	
	I find the purchase of nutritional food for pets inconvenient.	
Health risk (HR)	Additives used in pet nutritional food could adversely affect health.	(Choi et al., 2013; Huo & Liu, 2022; Zhang et al., 2024)
	Low-quality ingredients in pet nutritional food could have negative health implications.	
	Unsanitary production processes in pet nutritional food could pose HRs.	
Economic risk (ER)	I am concerned about whether the amount paid for pet nutritional food corresponds to its value.	
	I doubt whether I have purchased pet nutritional food at an appropriate price.	
	I worry that purchasing pet nutritional food could be a waste of money if ineffective.	
Social Risk (SR)	People around me do not understand my use of pet nutritional food.	
	People around me do not support my use of pet nutritional food.	
	People around me think using pet nutritional food is not a good idea.	
	I am concerned that using pet nutritional food could affect my image.	
Health Consciousness (HC)	I believe living in optimal health is very important for pets.	(Chen & Lin, 2018)
	I believe proper diet and preventive measures help maintain pet health.	
	I believe the health of pets depends on how they are cared for.	
	I actively strive to prevent diseases in pets.	
Attitudes (ATT)	I believe purchasing pet health food is wise.	(Ajzen, 1991)
	I believe purchasing pet health food is advisable.	
	I believe purchasing pet health food is beneficial.	
Continuous Purchase Intention (CPI)	I intend to continue purchasing pet nutritional food in the future.	(Y. Khan et al., 2022)
	I will frequently purchase pet nutritional food in the future.	
	I plan to increase the frequency of purchasing pet nutritional food.	

**Table 3:** Common method bias analysis.

Construct	Indicator	Substantive Factor Loading (R1)	R1^2^	Method Factor Loading (R2)	R2^2^
PSUS	PSUS1	0.838 ***	0.702	0.06 *	0.004
	PSUS2	0.757 ***	0.573	−0.003	0.000
	PSUS3	0.797 ***	0.635	−0.096 **	0.009
	PSUS4	0.831 ***	0.691	0.033	0.001
PSEV	PSEV1	0.823 ***	0.677	−0.022	0.000
	PSEV2	0.803 ***	0.645	−0.06	0.004
	PSEV3	0.805 ***	0.648	0.012	0.000
	PSEV4	0.827 ***	0.684	0.067 *	0.004
PB	PB1	0.826 ***	0.682	−0.058	0.003
	PB2	0.848 ***	0.719	0.035	0.001
	PB3	0.832 ***	0.692	0.021	0.000
PBA	PBA1	0.824 ***	0.679	−0.052	0.003
	PBA2	0.826 ***	0.682	0.027	0.001
	PBA3	0.838 ***	0.702	0.025	0.001
HR	HR1	0.870 ***	0.757	−0.032	0.001
	HR2	0.850 ***	0.723	−0.002	0.000
	HR3	0.815 ***	0.664	0.037	0.001
ER	ER1	0.804 ***	0.646	−0.046	0.002
	ER2	0.822 ***	0.676	0.019	0.000
	ER4	0.795 ***	0.632	0.027	0.001
SR	SR1	0.843 ***	0.711	−0.09 **	0.008
	SR2	0.778 ***	0.605	0.12 **	0.014
	SR3	0.717 ***	0.514	−0.024	0.001
	SR4	0.864 ***	0.746	0.002	0.000
HC	HC1	0.834 ***	0.696	−0.02	0.000
	HC2	0.782 ***	0.612	0.062	0.004
	HC3	0.764 ***	0.584	0.008	0.000
	HC4	0.737 ***	0.543	−0.052	0.003
ATT	ATT1	0.859 ***	0.738	−0.042	0.002
	ATT2	0.870 ***	0.757	−0.043	0.002
	ATT3	0.824 ***	0.679	0.087	0.008
CPI	CPI1	0.852 ***	0.726	−0.077 *	0.006
	CPI2	0.822 ***	0.676	0.102 **	0.010
	CPI3	0.849 ***	0.721	−0.024	0.001
Mean			0.671		0.003
Ratio			238		

* *p* < 0.05, ** *p* < 0.01, *** *p* < 0.001.

**Table 4:** Confirmatory factor analysis and reliability analysis.

Variable	Items	Items Loadings	Cronbach’s Alpha	CR	AVE
PSUS	PSUS1	0.845	0.820	0.881	0.649
	PSUS2	0.757			
	PSUS3	0.778			
	PSUS4	0.840			
PSEV	PSEV1	0.820	0.831	0.887	0.663
	PSEV2	0.792			
	PSEV3	0.806			
	PSEV4	0.838			
PB	PB1	0.805	0.783	0.873	0.697
	PB2	0.859			
	PB3	0.839			
PBA	PBA1	0.847	0.773	0.868	0.687
	PBA2	0.815			
	PBA3	0.824			
HR	HR1	0.880	0.800	0.882	0.714
	HR2	0.853			
	HR3	0.801			
ER	ER1	0.813	0.733	0.849	0.651
	ER2	0.824			
	ER4	0.784			
SR	SR1	0.860	0.814	0.877	0.643
	SR2	0.757			
	SR3	0.778			
	SR4	0.840			
HC	HC1	0.832	0.785	0.861	0.608
	HC2	0.801			
	HC3	0.766			
	HC4	0.716			
ATT	ATT1	0.855	0.810	0.888	0.725
	ATT2	0.870			
	ATT3	0.828			
CPI	CPI1	0.845	0.793	0.878	0.707
	CPI2	0.835			
	CPI3	0.842			

**Table 5:** Discriminant validity by Fornell-Larcker criterion.

	PSUS	PSEV	PB	PBA	HR	ER	SR	HC	ATT	CPI
PSUS	**0.806**									
PSEV	0.392	**0.814**								
PB	0.333	0.303	**0.835**							
PBA	−0.216	−0.229	−0.391	**0.829**						
HR	−0.340	−0.358	−0.324	0.203	**0.845**					
ER	−0.338	−0.368	−0.390	0.341	0.254	**0.807**				
SR	−0.329	−0.367	−0.361	0.346	0.388	0.378	**0.802**			
HC	0.318	0.359	0.336	−0.261	−0.407	−0.308	−0.376	**0.780**		
ATT	0.495	0.490	0.531	−0.443	−0.456	−0.544	−0.469	0.546	**0.851**	
CPI	0.317	0.364	0.305	−0.254	−0.333	−0.362	−0.335	0.365	0.654	**0.841**

The values along the diagonal line represent the square root of AVE.

**Table 6:** Results of SEM analysis.

Hypothesis	Path Coefficient (β)	T-Value	*p*-Value	Result
H1. PSUS → ATT	0.161	4.892	0.000 ***	Accept
H2. PSEV → ATT	0.127	3.722	0.000 ***	Accept
H3. PB → ATT	0.172	5.116	0.000 ***	Accept
H4. PBA → ATT	−0.141	4.360	0.000 ***	Accept
H5. HR → ATT	−0.106	3.190	0.001 **	Accept
H6. ER → ATT	−0.211	5.431	0.000 ***	Accept
H7. SR → ATT	−0.052	1.469	0.142	Reject
H8. HC → ATT	0.227	6.185	0.000 ***	Accept
H9. ATT → CPI	0.654	24.050	0.000 ***	Accept

** *p* < 0.01, *** *p* < 0.001.

**Table 7:** Results of predictive relevance using PLSpredict.

		RMSE		PLS-SEM—LM
Items	Q² Predict	PLS-SEM	Linear Model	Difference
ATT	0.411	0.851	0.878	−0.027
ATT	0.424	0.817	0.838	−0.021
ATT	0.444	0.833	0.859	−0.026
CPI	0.137	1.151	1.195	−0.044
CPI	0.225	1.065	1.103	−0.038
CPI	0.166	1.088	1.121	−0.033

**Table 8:** RMSE values.

	Training			Testing			
Neural Network	N	SSE	RMSE	N	SSE	RMSE	Total Samples
1	459	4.638	0.101	47	0.573	0.110	506
2	455	4.963	0.104	51	0.434	0.092	506
3	466	4.808	0.102	40	0.382	0.098	506
4	461	4.650	0.100	45	0.375	0.091	506
5	467	4.577	0.099	39	0.334	0.093	506
6	463	4.826	0.102	43	0.234	0.074	506
7	458	4.565	0.100	48	0.250	0.072	506
8	450	3.990	0.094	56	0.656	0.108	506
9	480	5.313	0.105	26	0.304	0.108	506
10	466	4.429	0.097	40	0.350	0.094	506
	Mean	4.676	0.100		0.389	0.094	
	SD	0.329	0.003		0.127	0.013	

Note: SSE: sum square of errors; RMSE: root mean square of errors; N: sample size.

**Table 9:** Sensitivity analysis.

Neural Network	PSUS	PSEV	PB	PBA	HR	ER	HC
1	0.164	0.216	0.093	0.125	0.152	0.159	0.091
2	0.090	0.164	0.124	0.159	0.119	0.173	0.171
3	0.142	0.130	0.117	0.143	0.110	0.198	0.161
4	0.132	0.121	0.121	0.129	0.116	0.195	0.185
5	0.117	0.090	0.133	0.132	0.158	0.135	0.234
6	0.153	0.163	0.122	0.103	0.132	0.155	0.172
7	0.132	0.133	0.145	0.119	0.129	0.182	0.160
8	0.128	0.117	0.139	0.108	0.147	0.180	0.181
9	0.114	0.097	0.132	0.111	0.114	0.244	0.187
10	0.144	0.126	0.150	0.104	0.135	0.147	0.194
Average importance	0.1316	0.1357	0.1276	0.1233	0.1312	0.1768	0.1736
Normalized importance (%)	74.43%	76.75%	72.17%	69.74%	74.21%	100%	98.19%

## Data Availability

All relevant data are available from the authors on request.
